# RGB‐Single‐Chip OLEDs for High‐Speed Visible‐Light Communication by Wavelength‐Division Multiplexing

**DOI:** 10.1002/advs.202404576

**Published:** 2024-10-24

**Authors:** Kou Yoshida, Cheng Chen, Harald Haas, Graham A. Turnbull, Ifor D. W. Samuel

**Affiliations:** ^1^ Organic Semiconductor Centre SUPA School of Physics and Astronomy University of St Andrews St Andrews KY16 9SS UK; ^2^ Electrical Engineering Division University of Cambridge 9 J J Thomson Avenue Cambridge CB3 0FA UK

**Keywords:** LiFi, optical wireless communication, organic light‐emitting diodes, wavelength‐division multiplexing

## Abstract

Organic light‐emitting diodes (OLEDs) have been developed for high‐speed transmitters of visible‐light communication (VLC) but so far the possibility of direct fabrication of multiple colors on a single substrate has not been exploited for multi‐Gbps data transmission. Very fast red‐, green‐, and blue (RGB)‐emitting OLEDs are developed on a single substrate to realize high data transmission speed by wavelength division multiplexing (WDM). −6 dB electrical bandwidth of over 100 MHz is achieved for all colors by selecting fluorescent materials with nanosecond emission lifetimes and little overlap between their emission spectra and incorporating them into OLEDs designed for high‐speed operation. Optical microcavities in top‐emitting OLED structures are used to minimize spectral overlap. A record data transmission rate for an OLED transmitter system of 3.2 Gbps is demonstrated, by transmitting data with the 3 colors simultaneously and separating each data by dichroic mirrors. The results show that WDM with integrated RGB pixels is a useful way to increase the data transmission rate of a VLC system based on OLED transmitters, which has the potential to enable multi‐gigabit transmission by displays. The availability of high‐speed multiple‐color devices as developed here also expands applications of OLEDs for spectroscopy, sensing, and ranging.

## Graphical abstract

High‐speed red, green, and blue organic light‐emitting diodes (RGB‐OLEDs) are integrated onto a single substrate and achieve a record data transmission rate for an OLED transmitter system of 3.2 Gbps by exploiting wavelength‐division multiplexing. It is demonstrated that integrating RGB pixels is a useful way to increase the data transmission rate of OLED transmitters.

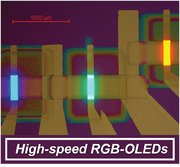

## 1 Introduction

Visible light communication (VLC) is of growing importance to address the significant increase in global demands for wireless communication bandwidth, as a complement to conventional radio frequency (RF) technologies used in Wi‐Fi and mobile phones. Because light consists of electromagnetic waves with much higher frequencies than those used in RF technologies, optical communication can potentially provide several hundreds of terahertz bandwidth, that is freely available. VLC is also less susceptible to interference than RF, and attractive for secure data communications.^[^ ^1^ ^]^ VLC can be combined with other functions such as illumination and displays, providing energy efficient and novel applications.^[^ ^2^ ^]^ Recently, a standard from the Institue of Electrical and Electronics Engineers (IEEE) for LiFi was approved (IEEE 802.11bb), which will accelerate commercialization and deployment of LiFi which initially is based on infrared. However, with the advent of future high‐bandwidth visible light devices, it is anticipated that future revisions will include VLC.

Organic light‐emitting diodes (OLEDs) are gaining interest as data transmitters for VLC to complement or compete with conventional inorganic LEDs due to their low‐cost fabrication, flexibility, and easy integration.^[^ ^1^, ^2^, ^3^, ^4^ ^]^ The use of OLEDs as high‐speed VLC transmitters has been limited because OLEDs were thought to be slow devices due to the low charge mobilities and large area and thin devices leading to high capacitance (*C*)^[^ ^1^, ^2^, ^4^ ^]^ as *C* = *εAd* ^−1^, where *ε* is the permittivity of the organic layers, *A* is the area, and *d* is the total thickness of organic layers. Reducing the active area can reduce the capacitance and this approach in combination with a high charge mobility polymer led to a relatively high −6 dB electrical bandwidth of 50 MHz.^[^ ^5^ ^]^ By combining small OLEDs with coplanar waveguide electrodes^[^ ^6^ ^]^ sub‐nanosecond optical pulses were demonstrated.^[^ ^7^ ^]^ We tackled the low charge mobilities by applying a high electric field while suppressing the thermal breakdown of OLEDs by using thermally conductive silicon substrates.^[^ ^8^ ^]^ We demonstrated very fast operation of OLEDs with a −6 dB electrical bandwidth of 245 MHz and a data transmission rate of 1.13 Gbps using an active area of 9 × 10^−4^ cm^2^. The OLED designs were further improved by Munshi et al. by reducing the active area and improving injection electrodes, to achieve a −6 dB electrical bandwidth of 459 MHz and a data transmission rate of 2.85 Gbps with an active area of 1.6 × 10^−5^ cm^2^.^[^ ^9^ ^]^ Through these recent advances, OLED speed and data transmission rate are getting closer to those of inorganic LEDs, which show −6 dB electrical bandwidth close to 1 GHz and a data rate of over 10 Gbps.^[^ ^10^ ^]^

Wavelength division multiplexing (WDM), in which multiple data streams are transmitted as different colors, is a powerful way to increase the data transmission rate. Often implementations of WDM involve multiple separate sources – different lasers or LEDs. Our goal was to simplify this using organic semiconductors which have the attractive feature that different colors can be deposited side by side. Different colors of OLEDs are individually modulated to transmit multiple data streams simultaneously. By tuning the sensitivity of photodiodes for different OLEDs, the data from each color can be successfully transferred and thus the data transmission rate increases linearly with the number of devices.^[^ ^11^ ^]^ Multicolor OLEDs can be directly fabricated on a single substrate – something widespread in the OLED displays of smartphones.^[^ ^12^ ^]^ This integration of colors can make a WDM transmitter based on OLEDs simple and compact. There are several attempts to implement a WDM scheme with OLED transmitters^[^ ^13^, ^14^, ^15^ ^]^ but so far a high‐speed WDM system based on OLED transmitters has not been demonstrated. Polymer OLEDs with three different colors were fabricated separately and showed an aggregated data rate of 54.9 Mbps by driving each color separately.^[^ ^14^ ^]^ Commercial blue‐ and red‐emitting OLEDs were used to transmit data together resulting in a total data rate of 140 kbps.^[^ ^15^ ^]^ More recently, single tricolor‐integrated OLEDs with a relatively large separation of 1 cm were developed and used to test interference by different colors of pixels at a limited data rate of 1 kbps for each channel.^[^ ^13^ ^]^

Here, we report the development of very fast red, green, and blue OLEDs (RGB‐OLEDs) and integrate all 3 pixels close together on a single substrate. Each pixel shows faster operation than the fluorescent lifetime of its emitter and we achieve record data transmission rates for red‐ and green‐emitting OLEDs. We construct a WDM system showing a record data transmission rate for an OLED transmitter by transmitting data with the 3 colors of OLEDs together and separating each emission with dichroic mirrors. The WDM system has a small reduction, 20%, in the data rate compared with the single systems. Thus, integrated RGB‐OLEDs is a promising format for high‐speed VLC.

## 2 Results and Discussion

### 2.1 Overview of Fast RGB‐OLED Design

**Figure 1:**
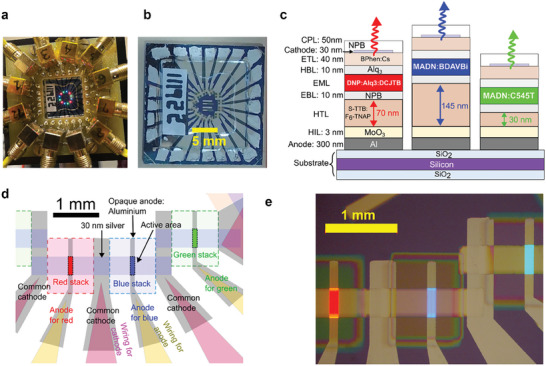
a) Photograph of the RGB‐OLED in the device holder operating all pixels together. b) Photograph of unmounted RGB‐OLEDs. Schematic c) cross‐section and d) top view. e) Microscope image under device operation. In part (e), the brightness of the image was uniformly increased for better visibility of the OLED contacts.

**Figure** **1** shows the overview of RGB‐OLEDs. Previously, we overcame the limitations of the electric time constant and transit time to make very fast blue fluorescent OLEDs which can be modulated as fast as the emitter with a photoluminescent (PL) lifetime of 1.1 ns.^[^ ^8^ ^]^ We realized this by minimizing electrical time constants with OLEDs with a small active area (9 × 10^−4^ cm^2^), 30 nm thick silver as transparent electrodes, and double wiring electrodes for the electrode and applying high voltage to increase charge velocity while suppressing thermal breakdown by using thermal conductive silicon substrates and p–i–n OLED structures. Now, we have developed fast green and red OLEDs by identifying green and red emitter materials with short emission lifetime^[^ ^13^, ^16^ ^]^ and incorporating them into our fast OLED design. We use 10‐(benzo[*d*]thiazol‐2‐yl)‐1,1,7,7‐tetramethyl‐2,3,6,7‐tetrahydro‐1*H*‐pyrano[2,3‐*f*]pyrido[3,2,1‐*ij*]quinolin‐11(5*H*)‐one (C545T) with a PL lifetime of 4.61 ns for the green OLEDs and 4‐(dicyanomethylene)‐2‐*tert*‐butyl‐6‐(1,1,7,7‐tetramethyljulolidin‐4‐yl‐vinyl)‐4*H*‐pyran (DCJTB) with 2.58 ns for the red OLEDs.

**Figure 2:**
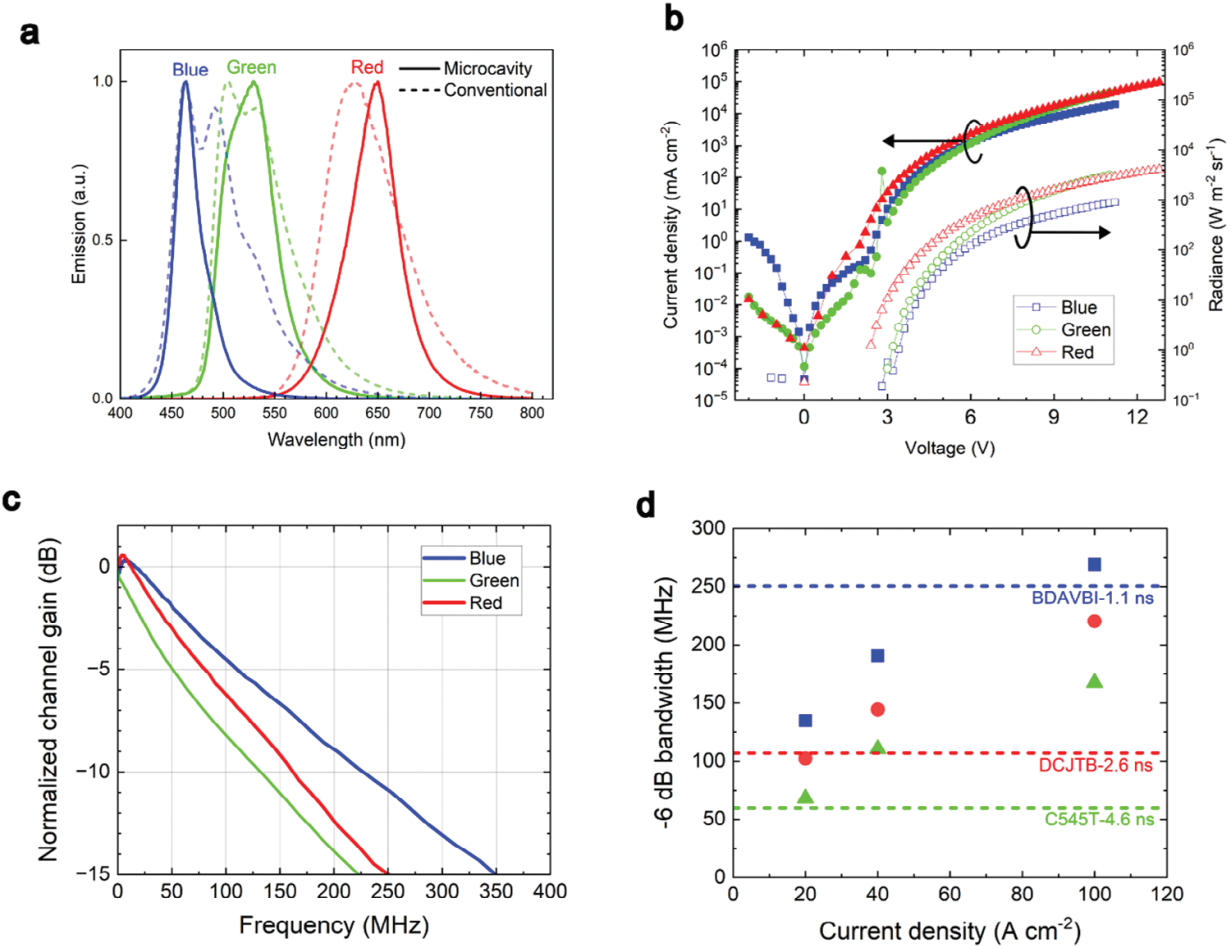
a) Comparison of EL spectra of RGB‐OLEDs with the top‐emitting microcavity structure (solid lines) and conventional bottom‐emitting structure with ITO (dashed lines). b) *J*–*V*–*R* characteristics, c) frequency response measured at DC offset of 20 A cm^−2^. d) −6 dB electrical bandwidth as a function of DC offset current density and comparison with the calculated bandwidth of emission layer films under optical excitation (dashed lines).

In WDM, the overlap of spectral characteristics of different channels should be minimized to avoid interference between different channels. Organic materials generally show broad electroluminescence (EL) spectra with full width at half maximum > 70 nm^[^ ^17^ ^]^ in conventional bottom‐emitting OLEDs based on indium tin oxide (ITO), as shown in **Figure** **2**a. This causes spectral overlap of emission from different colors of OLEDs especially for the blue and green OLEDs. We therefore incorporate the light emitting materials into an optical microcavity formed by a semitransparent silver cathode and an opaque aluminum anode to make a top‐emitting OLED. This gives much narrower spectra, and the emission can be tuned by changing the thickness of organic layers in the microcavity.^[^ ^13^, ^18^ ^]^ We adjusted only the thickness of the p‐doped hole transport layer (HTL, see Figure 1c) because this provides a simple fabrication process for making different colors of pixels together, while the high conductivity of the transport layer minimizes any increase of driving voltage with increasing the thicknesses. We chose HTL thicknesses of 70 nm for the red OLED, 30 nm for the green OLED, and 145 nm for the blue OLED. The red and green OLEDs used first order cavities while the blue OLED used a second order cavity. For top‐emitting OLEDs, the outcoupling efficiency of first order cavities is usually higher than second order cavities^[^ ^19^ ^]^ but we found thicker cavities were needed to make reliable blue devices. As shown in Figure 2a, microcavity OLEDs display relatively narrower spectra, and less overlap of each color compared with the conventional OLEDs. For example, the use of the microcavity reduced full width at half maximum of the emission from 72 to 28 nm for blue, from 75 to 58 nm for green, from 83 nm with the conventional OLED to 52 nm for red. We note that the emission spectra of our microcavity OLEDs blueshift slightly with angle, as shown in Figure S1a in the Supporting Information, leading to small changes of their spectral overlap.

Integrating many pixels of different colors of OLEDs is more challenging than fabricating single‐color OLEDs. There are several ways to construct multiple colors of OLEDs.^[^ ^12^ ^]^ We made them side by side by using shadow masks because of less restriction for materials and smaller reduction in performance compared with other methods. We used 7 different shadow masks (anode, cathode, wiring electrodes, blue, green, and red stacks, and common organic layers). The wiring electrode mask was required due to the mechanical flexibility of the 100 µm thick shadow masks, as shown in Figure S2 (Supporting Information). As the number of shadow masks which can be loaded into our evaporator was limited to 4, which was enough for the previous fast OLEDs,^[^ ^8^ ^]^ we had to break the vacuum of the evaporator chamber into a nitrogen atmosphere during the OLED fabrication. We initially did this just after HTL evaporation but found this resulted in speckles in EL images and poor current‐density voltage characteristics (see Figure S3 in the Supporting Information) due to the oxidation of the aluminum contact. We solved this by inserting MoO_3_ between the aluminum and the HTL without reducing the operational lifetime of the OLEDs.

As shown in Figure 1, the developed RGB‐OLED has different colors of OLED pixels located with a <1.5 mm separation (12 pixels in total on a single substrate), which is much closer than the previous WDM‐OLED work.^[^ ^13^ ^]^ The active area is around 5 × 10^−4^ cm^2^ (= 125 × 400 µm^2^). They are individually addressable by biasing individual anodes while sharing common cathodes. The ends of the pixels are brighter because the thickness of the thin silver is less there due to the shadowing effect for the shadow masks we used for the fabrication.

### 2.2 OLED Performance

Figure 2b shows current–voltage–radiance (*J*–*V*–*R)* characteristics of the RGB‐OLEDs. The RGB‐OLEDs can be operated at over 20 A cm^−2^ without device breakdown, which is much higher than typical OLEDs fabricated on a typical glass substrate, <1 A cm^−2^, due to the better thermal management with the silicon substrate.^[^ ^20^ ^]^ At 20 A cm^−2^, the red OLEDs reached a radiance of 1800 W m^−2^ sr^−1^ driving at 9.3 V, the green reached a radiance of 1900 W m^−2^ sr^−1^ driving at 9.5 V, and the blue reached a radiance of 920 W m^−2^ sr^−1^ driving at 10 V. The driving voltages for each color are similar to each other despite the blue OLEDs being much thicker, due to the doped HTL. The lower radiance of the blue OLED is due to light on the long wavelength side of the emission being suppressed by the microcavity to narrow down the emission spectra. We tested the operational stability of the blue and green OLEDs at 20 A cm^−2^ and the red OLED at 100 A cm^−2^, as shown Figure S1d (Supporting Information). The emission intensity dropped by 20% of its initial value under constant current operation for 4 min for the green, and 6 min for the blue and red OLEDs. Because the operating current density is much higher than the normal operating condition of OLEDs for displays and lighting, the operational lifetimes were much shorter than will be needed for practical use in VLC. We believe improving operational stability of our devices toward practical use should be possible with further developments because the operational stability of OLED has been improved many orders of magnitude over decades.^[^ ^21^ ^]^ We note that measurements of all EL characteristics measurements require less than a minute, so the short operational lifetime of our devices is not an issue for the measured results.

To test the speed of the OLED, the frequency response of an optical link was measured by modulating OLEDs under a DC offset current and detecting their emission with a high‐speed and flat response photodiode with a −6 dB electrical bandwidth from DC to 400 MHz^[^ ^22^ ^]^ (Thorlabs, APD430A2/M, see OLED characterization for more details). Figure 2c shows the normalized frequency responses of the OLEDs measured at a current density of 20 A cm^−2^. The channel gain drops as frequency increases due to the speed of the OLEDs limiting the speed of the link. The smaller reduction of the blue OLEDs compared with the red and green OLEDs showed that the blue OLEDs are faster than the other OLEDs. The −6 dB electrical bandwidth was determined at the frequency where the channel gain dropped by 6 dB from the value measured at 1 kHz. Figure 2d shows the bandwidth as a function of current density (see Figure S4 in the Supporting Information for the frequency response at different current densities). The bandwidth is observed to increase with current density and the bandwidths for the blue OLED are always the highest among the different colors and the bandwidth for green was the lowest at the same current density. This can be attributed to the longer PL lifetime of the green emitter limiting the response speed of the OLED. At 100 A cm^−2^, the bandwidth (averaged over 3 different pixels for each color) reached 255 MHz for the blue, 217 MHz for the red, and 171 MHz for the green. The red and green OLEDs have higher bandwidths than previously reported OLEDs in similar spectral ranges: <200 MHz for the red‐emitting OLEDs^[^ ^23^ ^]^ and 50 MHz for the polymer‐based green‐emitting OLEDs.^[^ ^5^ ^]^ We compared the response speed of the OLEDs with the response speed of the emitters in films under photoexcitation. PL lifetime is related to the PL bandwidth of the emitters (ƒ_6 dB‐PL_) as follows^[^ ^2^ ^]^

(1) $$f_{6\mathrm{dB}-\mathrm{PL}}=\frac{\sqrt{3}}{2\pi }\frac{1}{\tau_{\mathrm{PL}}}$$

where *τ*_PL_ is the PL lifetime. We used the reported the PL lifetime of the same emitter: 1.1 ns for 4,4‐bis[4‐(diphenylamino)styryl]biphenyl (BDAVBi),^[^ ^8^ ^]^ 4.61 ns for C545T,^[^ ^13^ ^]^ and 2.58 ns for DCJTB^[^ ^13^ ^]^ to calculate the PL bandwidths shown in Figure 2d. All OLEDs show a higher EL bandwidth at 100 A cm^−2^ than the corresponding PL bandwidth, showing that the OLEDs are operating in a regime in which the emission process itself can be a limiting factor in the speed of the OLEDs; in addition to charge transport or capacitive effects. The much higher bandwidth of the red and green OLEDs compared with the corresponding emitters under photoexcitation can arise from enhancement of the radiation process due to the Purcell effect in the optical microcavity^[^ ^24^ ^]^ including coupling to surface plasmon polariton modes and/or additional quenching processes in driving OLEDs such as quenching caused by triplet excitons.^[^ ^25^ ^]^ The microcavity effect increases PL bandwidth more than 1.6 times in the red and green OLEDs (Figure S5, Supporting Information). A further contribution to the increased bandwidth of the OLEDs can be expected as the external quantum efficiency (EQE) of the RGB‐OLEDs drops significantly at high current (Figure S1b, Supporting Information), indicating quenching that would shorten excited state lifetime and so increase bandwidth.

### 2.3 Data Communication Results

**Figure 3:**
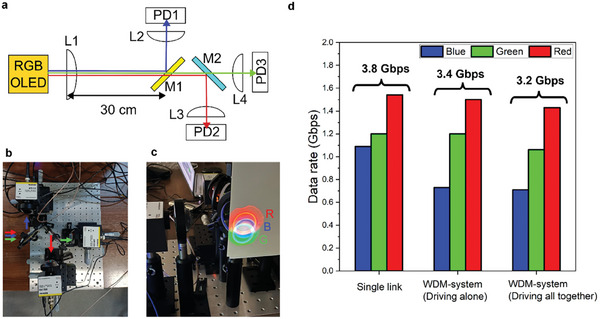
a) Schematic setup of WDM system. b) Photograph of the setup of the receiver side in the WDM system. c) Photograph of beams from RGB‐OLEDs projected in front of the first dichroic mirror (M1 in label (a)). d) Comparison of data rate of RGB‐OLED measured in different systems and different operating conditions.

We initially tested the data transmission rate of OLEDs of each color separately (single link, see Figure S6 in the Supporting Information). The light emitted by the OLED was collimated by a lens and then focused onto a photodiode (PD) at the receiver side by another lens. The positions of the collimator lens and the imaging lenses were optimized for each pixel color during single‐color testing. WDM measurements were performed in a similar way, but with two dichroic mirrors included to separate the different OLED colors to three receiver photodiodes, as shown in **Figure** **3**a,b. The optical setup was optimized to maximize the light intensity in each channel before the WDM measurements and fixed during the entire measurement. The link distance was set to 30 cm. We confirmed that the beam from each color of the OLED was transmitted together to the receiver side in the WDM system, as shown in Figure 3c. The ring shapes observed are due to the combination of optics used, not the emission of the OLEDs. Chaleshtori et al. provided a comprehensive review on modulation schemes and equalization schemes.^[^ ^1^ ^]^ Direct current biased optical orthogonal frequency division multiplexing (DCO‐OFDM) was used because it is more spectrally efficient than other modulation schemes such as on–off keying and allows us to compare directly with prior work using OLEDs^[^ ^8^, ^9^, ^15^, ^26^ ^]^ and inorganic LEDs.^[^ ^10^, ^11^ ^]^

Figure 3d summarizes the data rate measured for each color and configuration (see Figure S7 in the Supporting Information for the signal‐to‐noise ratio (SNR) spectra and Figure S8 in the Supporting Information for the bit error ratio (BER) as a function of the data rates). In “single link,” the data rates of the RGB‐OLEDs were 1.1 Gbps for the blue, 1.2 Gbps for the green, and 1.5 Gbps for the red. The data rates achieved for red and green channels are higher than for blue, even though the blue OLED has a higher bandwidth. This is because of the higher radiance and the better responsivity of the photodiodes used for the red and green channels compared with the blue channel. Our data rate of the red and green OLEDs is much higher than the previously reported OLEDs based on green‐ and red‐emitting polymers, which was lower than tens of Mbps.^[^ ^14^ ^]^

A slightly reduced date rate was found in the WDM system driving each pixel individually (driving alone) compared with the “single links,” as shown in Figure 3d. The obtained data rates were 0.73 Gbps for the blue, 1.2 Gbps for the green, and 1.5 Gbps for the red channels. Similar data rates of the red and reduced data rate of the blue compared with “single link” are reasonable considering the reflection spectra of the dichroic mirrors and the emission spectra of the OLEDs. 95% of photons emitting from the red OLED are expected to reach the photodiode for the red channel (PD2 in Figure 3a) and around 80% of the photons from the green and blue channel are expected to reach to each photodiode (PD1 for the blue and PD3 for the green, see Figure S9 and Table S1 in the Supporting Information). Similar performance of the green channel in the “WDM system and single links” is possibly due to a variation in the performance of the green pixel used for the measurements and the optical alignment.

Compared with “driving alone” data rates, small reductions of the data rate were observed when transmitting data in all channels simultaneously in the “WDM system” (driving all together). The obtained data rates were 0.71 Gbps for the blue, 1.1 Gbps for the green, and 1.4 Gbps for the red. We quantified the reduction due to crosstalk by calculating the ratio of the data rate “driving all together” to “driving alone.” The reduction of the data rate was 3% for the blue, 8% for the green, and 6% for the red. These small reductions means that crosstalk between the 3 channels was small. This validates our choice of emitter, use of microcavities, and selection of dichroic mirrors, enabling emission from each pixel to be separated effectively. An additional factor reducing crosstalk is that the small (<1.5 mm) spatial separation of each OLED pixel is larger than the size of each detector (<0.5 mm in diameter^[^ ^27^ ^]^) and so helps avoid receiving stray light from other pixels as occurs in a multiple‐input multiple‐output system based on an array of single‐color micro‐LEDs.^[^ ^28^ ^]^

## 3 Conclusion

We developed very fast and spectrally separated RGB‐OLEDs and integrated each pixel closely (<1.5 mm) on a single substrate. We demonstrated a record data rate for a VLC transmitter based on OLEDs by simultaneously transmitting data from the three different colors of the OLED by using wavelength‐division multiplexing. A data transmission rate of 3.2 Gbps at the 7% forward error correction (FEC) limit in a 30 cm data link was achieved. Compared to the previous record data transmission rate of 2.85 Gbps with a single OLED,^[^ ^9^ ^]^ we achieved a higher data in a similar link distance. WDM with RGB‐OLEDs is an effective way to increase data transmission rate with a small reduction of <20% in the total data rate compared with the aggregated data rates of the individual single links. As the reduction of the data rate in the WDM scheme is small, we believe that with improved data rate of individual OLEDs and more OLED colors, it will be possible to achieve over 10 Gbps data transmission with OLEDs. Our results suggest the potential of OLEDs for high‐speed short‐distance communication, such as display‐to‐display communication or device‐to‐device communication. Furthermore, our integrated multicolor OLEDs may be useful for other applications where spectral multiplexing, high brightness, and fast modulation are required such as spectroscopy and sensing.

## 4 Experimental Section

### RGB‐OLED Fabrication

The RGB‐OLEDs were fabricated by thermally evaporating materials through different custom‐made shadow masks (LiMaB GmbH, 100 µm thick) in a vacuum chamber at a base pressure of 10^−7^ mbar (Angstrom Engineering Inc., Evo Vac). To fabricate different colors of OLED stacks together, a part of a HTL, an electron blocking layer (EBL), an emission layer (EML), and a hole blocking layer (HBL) were evaporated individually through different “organic” masks for each color of stacks while the other layers were evaporated all color together. First, 200 nm thick silver as wiring electrodes and 300 nm thick aluminum as anodes were evaporated through the “wiring” mask and the “anode” mask, respectively, on precleaned highly resistive silicon substrates (upper resistivity 3000 Ω cm) and a thickness of 675 µm coated with 300 nm silicon dioxide layers on both sides (Inseto). Next, 3 nm thick MoO_3_ and a 30 nm 2,2′,7,7′‐tetrakis(*N*,*N′*‐di‐*p*‐methyl phenylamino)‐9,9′‐spirobifluorene p‐doped with 2,2′‐(perfluoronaphthalene‐2,6‐diylidene)dimalononitrile (4 vol%) as a HTL were evaporated through the “common organic” mask for all stacks, and then a further 40 nm HTL for the red stack and 115 nm HTL for the blue stack were evaporated. 10 nm *N*,*N′*‐di(naphthalene‐1‐yl)‐*N*,*N′*‐diphenylbenzidine (NPB) as EBL, EML, and 10 nm tris(8‐hydroxyquinolinato) aluminum (Alq_3_) as HBL were evaporated individually for each stack. The EML for the different stacks consisted of BDAVBi doped at 3 vol% in 2‐methyl‐9,10‐bis(naphthalen‐2‐yl)anthracene, 2‐methyl‐9,10‐di(2‐naphthyl)anthracene (MADN) with a thickness of 20 nm for the blue, C545T doped at 1.7 vol% in MADN with a thickness of 20 nm for the green, DCJTB at 1.5 vol% and Alq_3_ doped at 42 vol% in 3,9‐di(naphthalen‐2‐yl)perylene and 3,10‐di(naphthalen‐2‐yl) perylene mixture^[^ ^29^ ^]^ with a thickness of 14 nm for the red. 40 nm cesium‐doped 4,7‐diphenyl‐1,10‐phenanthroline as an n‐doped ETL, 30 nm silver layer as cathode, and 50 nm thick NPB capping layer were evaporated on all stacks together by using the common organic mask for the organic layers and the cathode mask. After the HTL evaporation of all stacks, the evaporator was vented to swap shadow masks. All OLEDs were encapsulated under a nitrogen atmosphere using 1.1 mm thick custom‐made glass lids with a cavity (Luminescence Technology Corp.), an epoxy glue (Norland Products Inc., Norland Optical Adhesive 68) prebaked under a nitrogen condition, and a moisture getter (Dynic Corporation, HD‐071210T‐50S). The active areas were measured from EL images of the operating OLEDs under a microscope (ECLIPSE LV100ND, Nikon).

A similar procedure was used to make conventional bottom‐emitting OLEDs based on the same emission layer on ITO as an anode and aluminum as a cathode. Precleaned 1.1 mm thick glass substrates coated with a 117 nm thick prepatterned ITO anode (Xin Yan Technology Ltd.) were applied to the oxygen plasma treatment, spin‐coated with poly(3,4‐ethylenedioxythiophene):polystyrene sulfonate (PEDOT:PSS, Heraeus Clevios, Al4800) diluted by deionized water (DIW) at 3000 rpm and then baked at 120 °C for 10 min. The ratio of PEDOT:PSS and DIW was 1:2 by volume. The PEDOT:PSS layer was used instead of MoO_3_ to prevent shorting. The organic layers were the same as the RGB‐OLED but with HTL and ETL thicknesses adjusted to give efficient outcoupling of light: 30 nm HTL and ETL for the blue, 30 nm HTL and 45 nm ETL for the green, and 35 nm HTL and 50 nm ETL for the red. The organic layer stacks were capped with 150 nm thick aluminum as anode.

### OLED Characterization

Current density–voltage–radiance characteristics were tested with a source measure unit (Keithley Instruments, Keithley 2400) and a calibrated silicon photodiode module reading out its output voltage with a multimeter (Keithley Instruments, Keithley 2000). The maximum scanning voltage of different OLEDs prior to the communications experiments was limited. External quantum efficiency and power efficiency of the OLEDs were calculated by measuring light emitted in the forward direction and considering the emission pattern of RGB‐OLEDs obtained by an optical simulation.^[^ ^30^ ^]^ A simulation was used because measuring the actual emission patterns of the OLEDs was difficult because their holder physically blocked light at more than 50°. The simulation was based on a model that treated the emission dipole as a forced‐damped harmonic oscillator embedded in a thin‐film stack.^[^ ^30^ ^]^ The HTL thickness in the simulation was fitted to reproduce the experimental angular resolved spectra, as shown in Figure S1a (Supporting Information). The fitted HTL thicknesses were 140 nm for the blue OLED, 28 nm for the green OLED, and 66 nm for the red OLED. Figure S1e (Supporting Information) shows the calculated emission pattern of RGB‐OLEDs. Emission spectra were measured with a calibrated fiber‐coupled spectrograph (MS125, Oriel) equipped with a charge‐coupled device camera (DV420‐BU, Andor). The emission spectra were recorded by placing the fiber normal to the substrate surface of OLEDs and driving them at constant current density, 20 A cm^−2^ for the RGB‐OLEDs and 10 mA cm^−2^ for the conventional OLEDs.

The frequency responses of the OLEDs were tested using a fast and flat frequency response photodiode (Thorlabs, APD430A2, −3 dB bandwidth of DC to 400 MHz). A pair of lenses (Thorlabs, ACL5040U‐A) was used to collect emission from the OLEDs and imaged onto the photodiode. The OLEDs and the photodiodes were directly connected to a vector network analyzer (Keysight, E5061B): the OLEDs for port 1 and the photodiodes for port 2. The signal transmission coefficients “S21” were recorded at frequencies in the range of 1 kHz to 1 GHz and different voltages with a fixed RF power of −6 dBm. Current density was measured by the network analyzer. The measured channel gain was normalized at 1 kHz. The output voltage of the photodiode was checked with a 500 MHz oscilloscope (Tektronix, TDS3052C) and the gain of the photodiode was adjusted to avoid saturation of the photodiode, which could change the frequency response.

### VLC Link Setup

The RGB‐OLEDs were mounted on the custom‐made holder, as shown in Figure 1a. A heat sink with a fan was equipped with the OLED to dissipate Joule heating at high driving conditions. Data rates were measured under typical office illumination levels, which included natural light from windows in combination with artificial light from fluorescent lamps. Random digital signals were generated and modulated to the DCO‐OFDM signals by custom‐made computer software based on MATLAB (MathWorks). An adaptive bit loading, and power loading algorithm based on the work by Campello was used to achieve optimized experimental results.^[^ ^31^ ^]^ A fast Fourier transform size of 1024 was used for the OFDM signals and a cyclic prefix of 20. A clipping level of 3.2 was set. Examples of bit loading results for the red OLED in the single channel were presented in Figure S10 (Supporting Information). The signals were passed onto an arbitrary waveform generator (AWG, Keysight, M8195A) with analog output. The signal created by the AWG was amplified with a broadband amplifier (Mini‐Circuits, ZHL‐4240w+) and combined with a DC offset voltage from a power supply (Keysight, E36313A) through a bias tee (Mini‐Circuits, ZFBT‐4R2GW+) The output signals from the bias tee were applied to the OLEDs. All data rates were tested at a bias current density of 20 A cm^−2^, which was controlled by the power supply. The value of current density was similar as the best condition found in the previous work.^[^ ^8^ ^]^ The peak‐to‐peak voltage of the OFDM signal set onto the arbitrary form generator was 75 mV for the blue and the red OLEDs and 80 mV of the green OLEDs.

Figure 3 shows the optical setup used in the WDM system. An aspheric lens (L1 in Figure 3a, Thorlabs, ACL7560U‐A) was used to collimate the output lights of the OLEDs together. A long‐pass dichroic mirror with a cut‐on wavelength of 490 nm (M1, Thorlabs, DMLP490L) was placed 30 cm away from lens L1 and used to reflect the signal from the blue OLEDs while passing the lights from red and green OLEDs. Also, a short‐pass dichroic mirror with a cutoff wavelength of 567 nm (M2, Thorlabs, DMSP567L) was put after mirror M1 to separate the red‐OLED emission from the green OLED. Each color of separated light was focused onto high bandwidth photodiode modules with aspheric lenses (L2‐4, Thorlabs, ACL50832U‐A). APD410 (PD1, Thorlabs, 3 dB bandwidth of 5–1000 MHz) was used for the blue OLED and APD210 (PD2 and PD3, Thorlabs, 3 dB bandwidth of 5–900 MHz) was used for the green and red OLEDs. These photodiodes could detect light modulated at higher frequencies than the ones used for the bandwidth measurement which were limited to <500 MHz. Up to 750 MHz for data communication was used. The output from the photodiode modules was captured by a high‐speed oscilloscope (Keysight, MXR608A) and then processed using custom‐made software. In the “driving all together” condition, each color of pixel was transmitting different OFDM signals together but the data rate from each channel was evaluated individually. The link distance was defined as the distance between the collimator lens and the imaging lens for the “single link”, and the distance between the collimator lens and the first dichroic mirror for the “WDM system”.

Figure S8 (Supporting Information) shows the measured BER as a function of data rate for different OLEDs measured in different conditions. The data rates were compared at the 7% FEC limit, which corresponded to a BER of 3.8 × 10^−3^. Linear interpolation was used between the nearest higher and lower data points to extract the data rate.

The fraction of photons arriving at each PD from each color of OLED was calculated using the measured emission spectra of the RGB‐OLEDs, and the transmission and reflection spectra of the dichroic mirrors provided by the manufacturer.^[^ ^32^ ^]^

## Conflict of Interest

H.H. is a founder, director and shareholder of pureLiFi, a company developing LiFi technology. The other authors declare no competing interests.

## Supporting information



Supporting Information

## Data Availability

The data that support the findings of this study are openly available in the University of St Andrews Research Portal at https://doi.org/10.17630/4fdba42a-c0de-4df6-a7ba-1614ad038308.
