# Supplementary material for: RGB‐Single‐Chip OLEDs for High‐Speed Visible‐Light Communication by Wavelength‐Division Multiplexing
Source: Adv Sci (Weinh). 2024 Oct 24;11(47):2404576. doi: 10.1002/advs.202404576 (PMC11653681; doi:10.1002/advs.202404576)
Supplement: Supplementary file 1 — Supporting Information [file ADVS-11-2404576-s001.pdf]

ADVANCED SCIENCE
Open Access

Supporting Information

for *Adv. Sci.*, DOI 10.1002/advs.202404576

RGB-Single-Chip OLEDs for High-Speed Visible-Light Communication by Wavelength-Division Multiplexing

Kou Yoshida, Cheng Chen, Harald Haas, Graham A. Turnbull* and Ifor D. W. Samuel**

Supporting Information

RGB-single-chip OLEDs for high-speed visible-light communication by wavelength- division multiplexing

Kou Yoshida, Cheng Chen, Harald Haas, Graham A. Turnbull*, and Ifor D. W. Samuel**

Figure S1

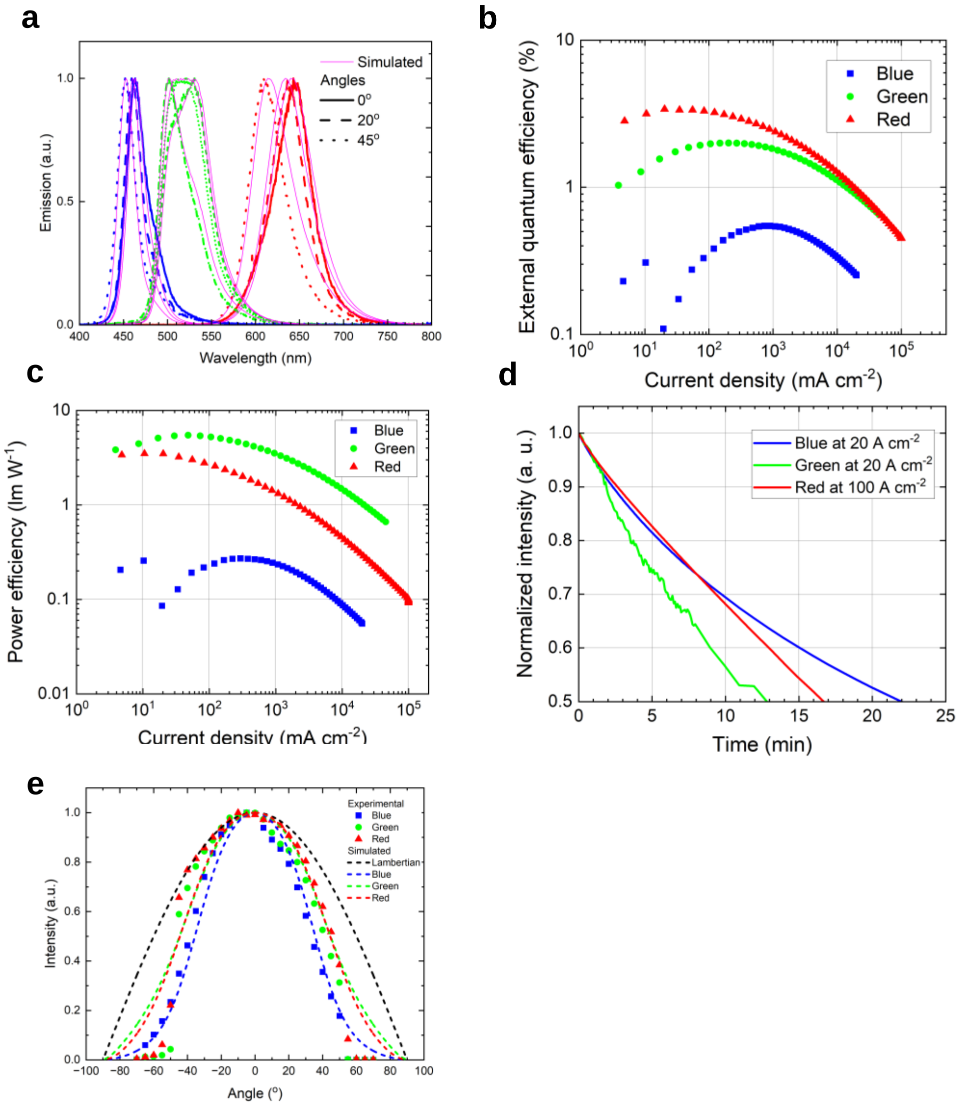

Figure S1. (a) Angle-resolved emission spectra, (b) current density - external quantum efficiency and (c) current density - power efficiency characteristics of RGB-OLEDs. (d) Emission intensity as a function of time of RGB-OLEDs at constant current operation. (e) Spectrally integrated radiant intensity as a function of the angle normalized to intensity in forward direction. For (a), we note our OLED holder physically blocks light at more than 50° so we show the results over a limited range of angles. The lens we used to collimate the OLED emission, ACL7560, has a numerical aperture, NA of 0.618, and so collects light at angles up to 40°. This means the measured spectra cover the relevant angular range in our VLC measurements. In part (d), the blue and green OLEDs were operated at 20 A cm^{-2} and the red OLED was operated at 100 A cm^{-2}. The emission intensity dropped by 20% of the initial value after constant current operation for 4 mins for green and 6 mins for blue and red.

Figure S2.

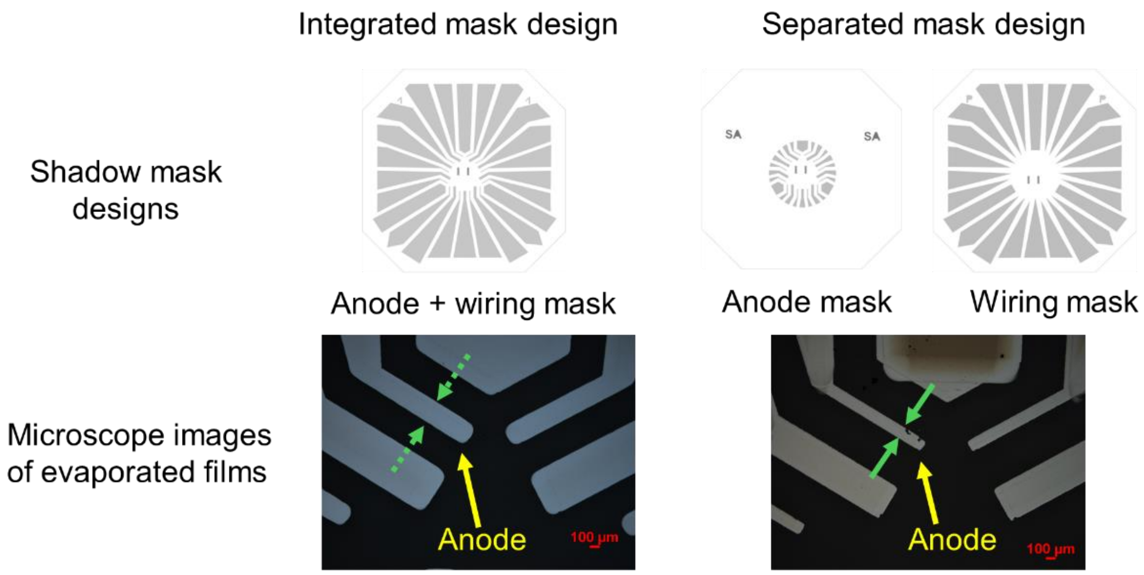

Figure S2. Comparison of different shadow mask designs for the anode of RGB-OLEDs and microscope images of evaporated films made using them. Integrated masks cause broader features than designed while separate masks produce consistent and similar features.

Figure S3

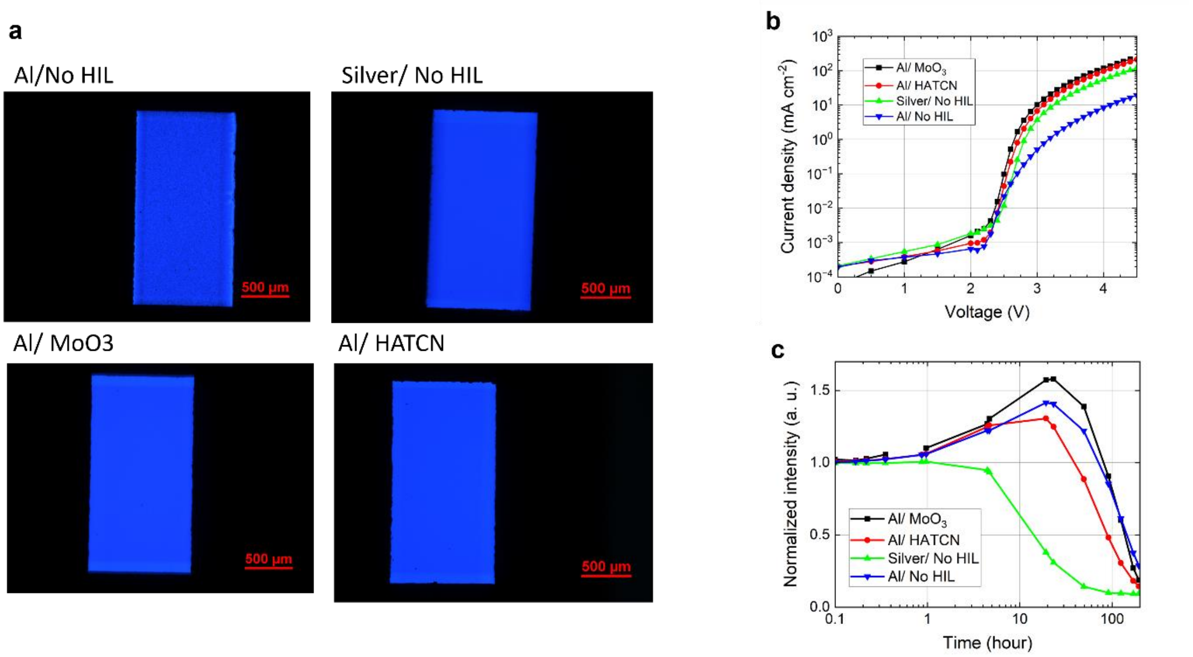

Figure S3. Microscope images of top-emitting OLEDs with different anode and different hole injection layers (HILs). (b) *J*-*V* characteristics. (c) Operational lifetime measured at a constant current density of 200 mA cm^{-2}. The device structure is similar to the blue OLEDs except for the thickness of HTL of 150 nm and bis(8-hydroxy-2-methylquinoline)-(4-phenyl phenoxy)aluminium (BAlq) was used as HBL. In part (a), the brightness of the photos was uniformly increased for the visibility of speckles in EL images. Without HIL, Al anode oxidized when we exposed the OLED stack to a nitrogen atmosphere after HTL evaporation. This resulted in speckles in EL images and poor *J*-*V* characteristics. These were suppressed by using a silver anode or by inserting HILs, 5 nm thick MoO_3 or 5 nm thick 1,4,5,8,9,10-hexaazatriphenylenehaxacarbonitrile (HATCN).

Figure S4

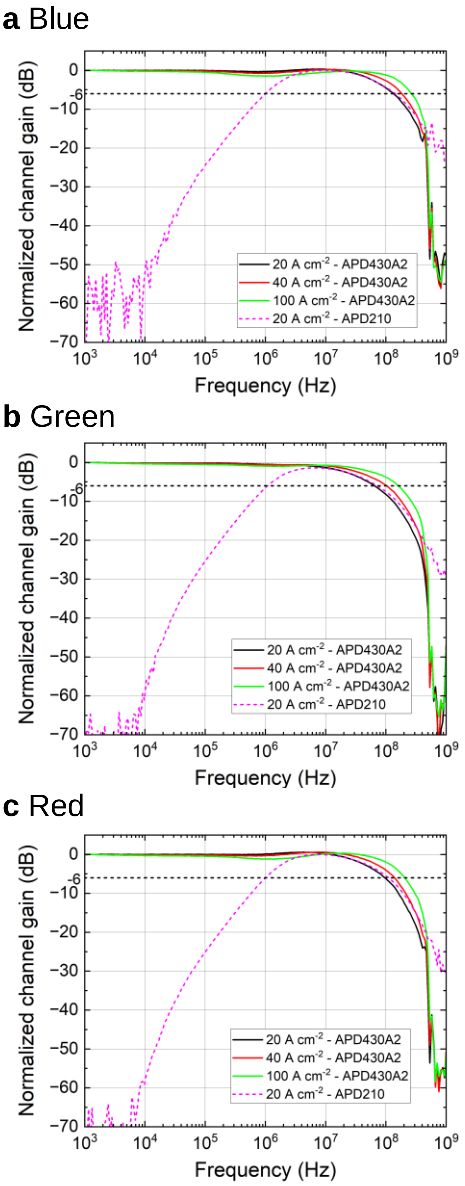

Figure S4. Frequency response of the RGB-OLED measured at different DC offset current densities and with different photodiodes by using the network analyzer. With APD430A2, the response was flat over a wide frequency range from 1 kHz to 10 MHz and significantly dropped above 500 MHz. With APD210, roll-up in channel gain was observed with a maximum at around 10 MHz and a decrease up to 1 GHz. These responses are due to the responses of the different photodiodes. Based on specifications, APD430A2 has a -3 dB bandwidth of from DC to 400 MHz[22] and APD210 has 5MHz to 1 GHz[27]. Due to the flat response over the wide frequency range from low to relatively high frequency, APD430A2 was used to determine the -6 dB bandwidth of OLEDs, while APD210 was used for data communication due to the availability of higher frequency (>500 MHz).

Figure S5

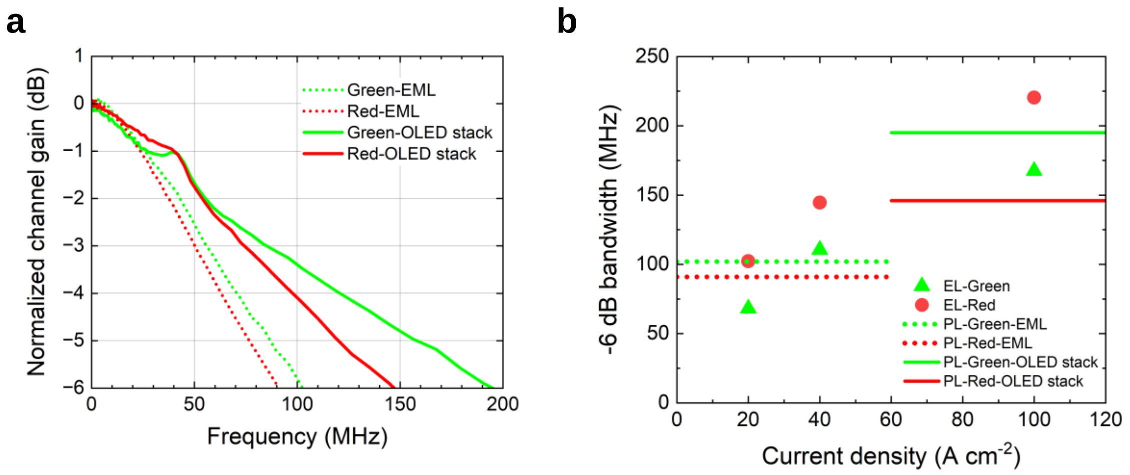

Figure S5. (a) PL frequency responses of the emission layer films and the OLED stacks of the green and red OLEDs. (b) Comparison of the PL and EL bandwidths. We used a 450 nm laser diode for excitation and a 495 nm long-pass filter in front of the detector. We considered the frequency response of the laser to obtain the measured PL frequency response. We note that the blue-OLED stack is difficult to measure because many layers in the OLED stack are excited with an excitation laser for the blue emission layer and their emission spectra are overlapped with the blue emitter.

Figure S6

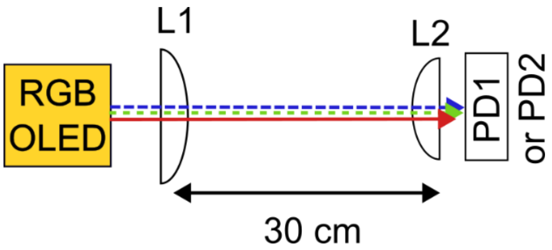

Figure S6. Schematic set-up of single link system

Figure S7

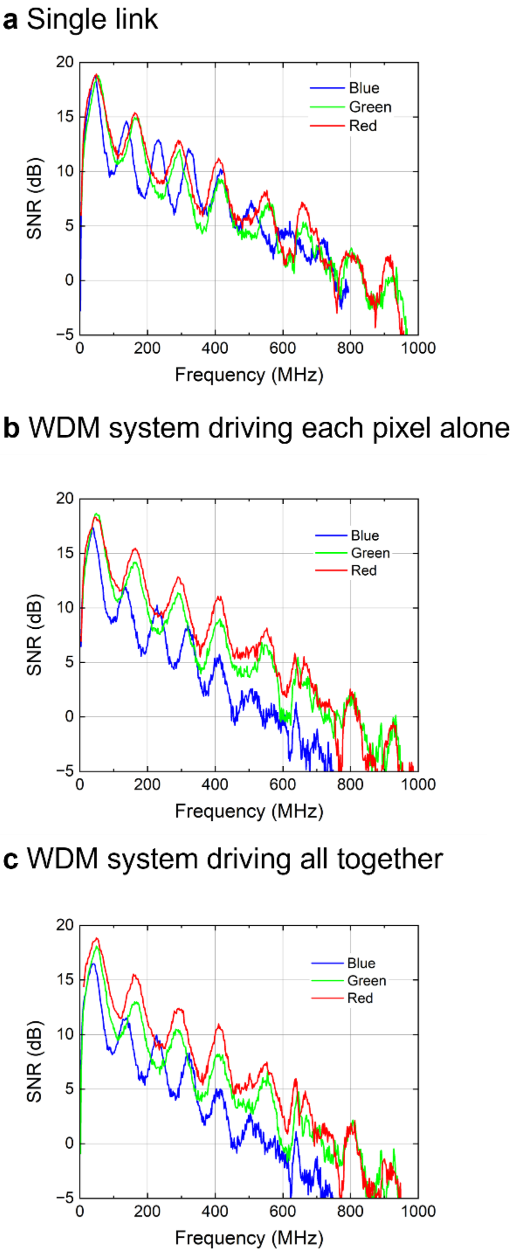

Figure S7. SNR spectra of the RGB-OLED measured by changing data rate in different configurations: (a) Single link, (b) Driving each pixel alone in the WDM system, and (c) Driving all pixels together in the WDM system.

Figure S8

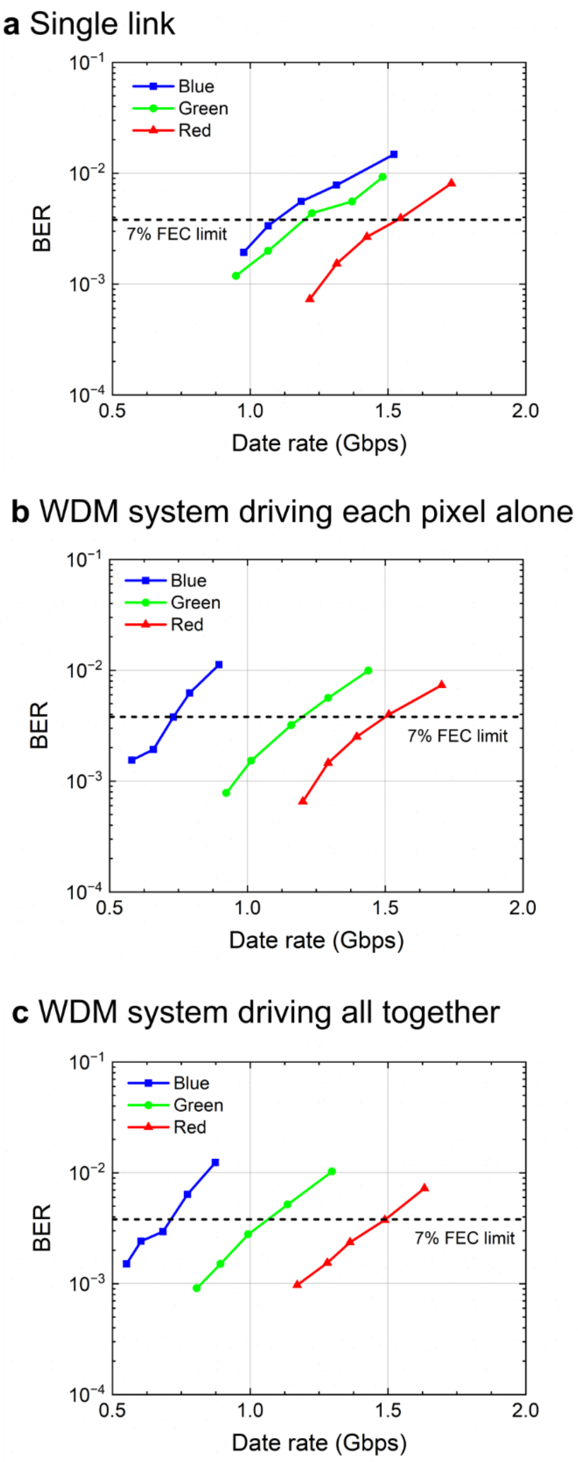

Figure S8. Bit error ratio (BER) as a function of data rate of the RGB-OLED measured by changing data rate in different configurations: (a) Single link, (b) Driving each pixel alone in the WDM system, and (c) Driving all pixels together in the WDM system.

Figure S9 and Table S1

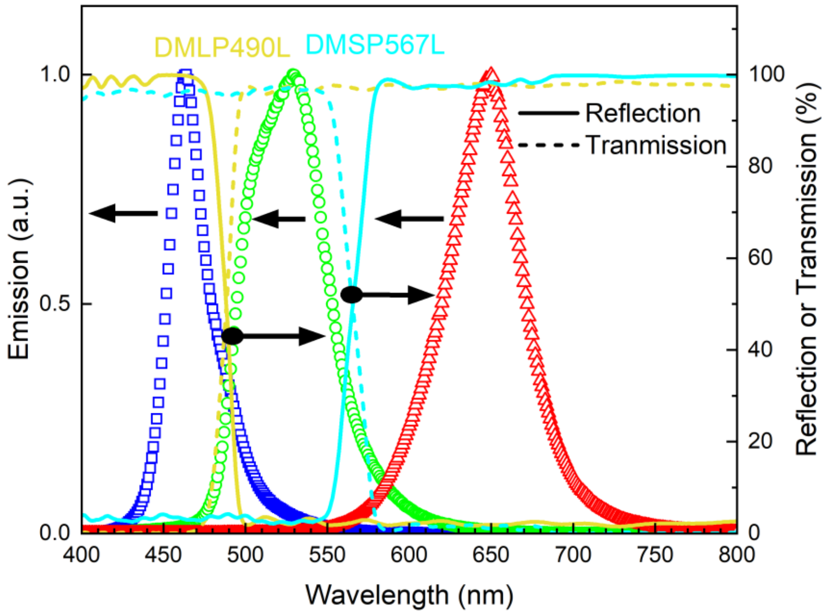

Figure S9. Comparison of EL spectra of the RGB-OLEDs (symbols) and reflection (solid lines) and transmission (broken lines) spectra of the dichroic mirrors used in the WDM system. The reflection and transmission data of the mirrors were obtained from ref. [32].

Table S1. Calculated fraction of photons arriving at each PD from each color of OLED.

	Blue	Green	Red
PD1	80%	6%	2%
PD2	1%	13%	95%
PD3	18%	79%	2%

Figure S10

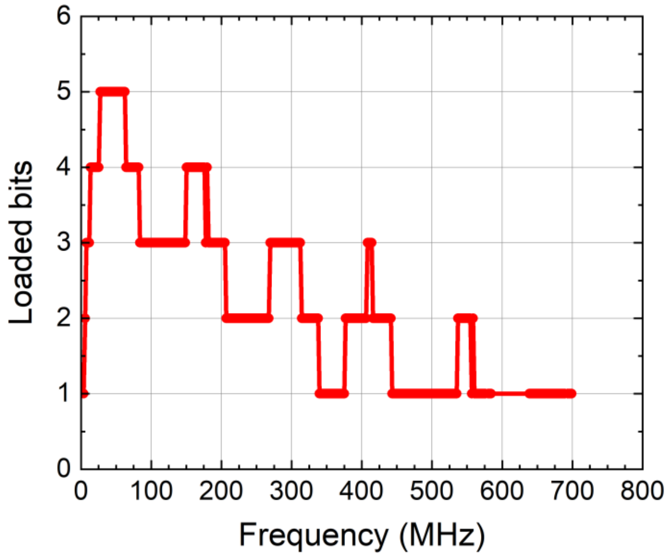

Figure S10. Bit loading results for the red OLED in the single link with data rate 1.42 Gbps and BER of $2.7 x 10^{-3}$. The modulation bandwidth is 700 MHz.
